# From “What Is?” to “What Isn't?” Computational Biology

**DOI:** 10.1371/journal.pcbi.1004318

**Published:** 2015-07-02

**Authors:** Ruth Nussinov, Sebastian Bonhoeffer, Jason A. Papin, Olaf Sporns

**Affiliations:** 1 Cancer and Inflammation Program, Leidos Biomedical Research, Inc., Frederick National Laboratory for Cancer Research, National Cancer Institute, Frederick, Maryland, United States of America; 2 Sackler Institute of Molecular Medicine, Department of Human Genetics and Molecular Medicine, Sackler School of Medicine, Tel Aviv University, Tel Aviv, Israel; 3 Theoretical Biology Group, Institute of Integrative Biology, ETH Zurich, Zurich, Switzerland; 4 Department of Biomedical Engineering, University of Virginia, Charlottesville, Virginia, United States of America; 5 Department of Psychological and Brain Sciences, Indiana University, Bloomington, Indiana, United States of America

This year, *PLOS Computational Biology* is celebrating its 10th birthday. Such a milestone provides an excellent occasion to reflect on the transformation that the field has undergone during the journal’s lifetime, and on the challenging question of where we may expect it to go next. As the leading journal in computational biology, *PLOS Computational Biology* encompasses the entire discipline and is therefore well placed to narrate this remarkable story. The evolution of the journal tells a rewarding story of success and accomplishment.

Computational biology has forged ahead, branching into every field of the biological sciences, and has become an integral part of basic biological research from molecules to ecosystems. We are no longer asking, "What is computational biology?” Instead, glancing over recent publications in *PLOS Computational Biology*, a more apt question is, “Is there any area of biology that doesn’t involve computational biology?” Indeed, nowadays most life science departments search for and hire faculty whose research embraces sophisticated approaches to computational biology. Increasingly, graduate training programs include computational modeling and data analysis as integral parts of the curriculum. This reflects not only the scientific merit of a mature field; it also points to a wider recognition that a background in computational biology is becoming mandatory. Students need to have solid training in computations.

What will we be able to accomplish in the next ten years that we were not able to achieve over the last ten? The explosion in computational biology has been driven by the rapid increase in the availability of experimental data and computational power. Both were, and will continue to be, revolutionized; both promise to keep transforming cutting-edge computational biology. “Big data” is a recurring theme, and as the volume of data continues to increase, there will be a growing need for computational tools and techniques to convert “big data” into biological knowledge, a challenge supported by the National Institutes of Health via the BD2K grants launched in 2012.

Firstly, the next decade will very likely be more data-intensive than the last decade. Biological data will increasingly be quantitative, substituting and supplementing traditional, descriptive biology. Examples (chosen for illustrative purposes) include identifying, cataloging, and analyzing microbes populating different tissues, ages, and health states, as well as microbes residing in the soil or in the ocean in specific environments where they can be useful, for example, against oil spills; discovering and classifying parallel cellular pathways that activate overlapping (or distinct) functions, for example, those emanating from the same family of receptors that may take over in cancer; and unveiling the combinatorial post-translational modifications (PTMs) codes, in which distinct PTM combinations spell specific (though related) functions for the same protein. There are hundreds of PTM types; each has specific chemistry, shape, and unique structural consequences. The number of possible combinations is immense. For example, in transcription factor p53, there are at least 50 PTM sites; the FoxO family of forkhead transcription factors is regulated by specific combinations of PTMs, including phosphorylation, acetylation, and ubiquitylation, in which distinct FoxO PTM combinations act as a “FoxO code.” Seventeen possible PTM acceptor residues were described in FOXO3a (Forkhead box O3) alone, and it was estimated that single and binary multiple modifications could give rise to thousands of different PTM isoforms. The vast amounts of data span ecosystems, diagnostic codes, diverse tumor clone phenotypes, and mapped mutations—single as well as combinations in which each mutation on its own has not been observed to drive a phenotypic change but together they do—can open new horizons and permit new analyses not possible before. When bolstered by a repertoire of patient-centered data, they may lead to more informed, knowledge-based correlations of physiological traits and predictive disease markers.

At the same time, data on such huge scales will also require development of methods to derive meaningful conclusions that are robust with respect to measurement error and noise, inherent biological variability and statistical biases. We anticipate that data accuracy and consistency will be major hurdles that will need to be overcome. These include background effects, noise such as that resulting from expression levels, which is mostly Poisson-like for high expression levels but more complex at low expression levels. Other sources of noise include differential binding strengths for different probe-target combinations; for example, the brightness of a spot depends on the binding strength between the probe and the target, which is itself a function of the specific sequence at the binding site. Noise sources also include lack of observed correlation between mRNA levels and protein levels, which may be due to the fact that some proteins are regulated after translation, insufficient spatial resolution, and more. These problems are compounded by lack of standardized procedures across experiments, which may lead to different levels of random noise, unsynchronized cell cycles in the sample and tissue inhomogeneity, which may obscure cell-specific data. In addition to noise induced by measurement error, the inherent variability and rich structure of biological systems poses challenges for data acquisition and analysis. These challenges are paramount in mapping structure and function of complex systems such as the brain.

Capturing population heterogeneity and associating it with function, whether for single molecules or for cells, poses yet another special challenge. One example of the multitude in this category is how to model the heterogeneous three-dimensional genome population across cell diversity, time, and space based on data including genomic sequences, function, and epigenetic information. This presents a daunting goal for the next ten years, but one that would have been virtually impossible when *PLOS Computational Biology* launched.

Computational neuroscience gives us another key example of how computational methods are transforming the traditional landscape of the biological sciences.

Current neuroscience databases can provide information relating to gene expression, neurons, macroscopic brain structure, and neurological or psychiatric disorders. Some databases contain descriptions of neuronal morphologies or interconnections, some record the activity of neurons or brain regions in relation to behavioral or cognitive functions, and others comprise volumetric imaging data, such as stacks of postmortem brain sections or 3-D MRI and fMRI images. This cornucopia of data is helping scientists understand and model the structure and function of the brain, widely recognized as one of the most challenging scientific frontiers of the 21st century. As the number of neuroinformatics resources that seek to disseminate information about the structure, development, and function of the brain has grown, so has the need to exploit them to advance basic scientific insight. Development of capable computational methods that can be applied across diverse types of data is critical and forms an integral part of computational (neuro)biology. These require not only advanced computing power; they also increasingly demand integration of data across different domains (e.g., genomics, physiology, behavior) and levels of biological organization (cells, circuits, systems) into a single analytic or modeling framework.

On their own, neither computations nor experiments can overcome these pressing challenges. Our current computational power can only simulate a small fraction of the time scale of biological processes, and it is limited to a tiny portion of the real molecular complex in a cellular environment. To mention just one example, the complete conformational change of the R to T transition in hemoglobin occurs in tens of microseconds. Using current resources, our large-scale simulations of a small protein can only account for processes covering microseconds. To effectively complement experimental studies, the time and spatial scales covered in computational biological models must continue to grow, beyond our current capabilities. Only exascale computing can meet the pressing challenges facing the biological sciences. Exascale computing, capable of at least one exaFLOPS, or a billion billion calculations per second, represents a 1,000-fold increase over the first 2008 petascale computer. While the National Institutes of Health is currently considering the possibilities of insights to be gained based on future computational resources, exploiting such power, estimated to become available in 2018 (at the earliest), may—under the best of circumstances—only be realized a decade from now.

Finally, *PLOS Computational Biology* is a community journal, championing the open access paradigm. Within this framework, we believe in data and software sharing. This has always been of paramount importance; with the unprecedented advancement of the field and its diversified disciplines, open data and software will become even more critical to drive further revolutions and to enlist participation from across the globe. No one knows what the future will bring—but it seems certain that the future is not going to be less computational; instead it is likely that computational analysis, tools, and models will become seamlessly integrated into the wider field of biology. In parallel with the inevitable expansion of biological data, computational biology will be indispensable for developing tools and approaches that are robust against noise and statistical biases, for devising testable and predictive models, and for delivering quantitative insight into fundamental biological processes within and across scales of organization. *PLOS Computational Biology* has been part of the field from its infancy, when questions such as, "What is computational biology?" were common, through to today, when, "What isn't computational biology?” seems far more appropriate. Together with the International Society for Computational Biology (ISCB) community and our broad and increasing readership, the journal will continue to serve as a forum for all aspects of computational biology, leading the discipline into its next decade.

